# Effects of Extraction Methods on Phenolic Content, Antioxidant and Antiplatelet Activities of Tomato Pomace Extracts

**DOI:** 10.3390/plants12051188

**Published:** 2023-03-06

**Authors:** Andrea Plaza, Lyanne Rodríguez, Anibal A. Concha-Meyer, René Cabezas, Elsie Zurob, Gastón Merlet, Iván Palomo, Eduardo Fuentes

**Affiliations:** 1 Centro de Estudios en Alimentos Procesados-CEAP, Conicyt, Programa Regional R19A10001, Gore Maule, Talca 3480094, Chile; 2 Thrombosis Research Center, Medical Technology School, Department of Clinical Biochemistry and Immunohematology, Faculty of Health Sciences, Universidad de Talca, Talca 3480094, Chile; 3 Instituto de Ciencia y Tecnología de los Alimentos, Facultad de Ciencias Agrarias y Alimentarias, Universidad Austral de Chile, Campus Isla Teja s/n, Valdivia 5090000, Chile; 4 Departamento de Química Ambiental, Facultad de Ciencias, Universidad Católica de la Santísima Concepción, Concepción 4070129, Chile; 5 Laboratory of Membrane Separation Processes (LabProSeM), Department of Chemical Engineering, University of Santiago de Chile, Santiago 9170022, Chile; 6 Departamento de Agroindustrias, Facultad de Ingeniería Agrícola, Universidad de Concepción, Chillán 4070386, Chile

**Keywords:** cardiovascular diseases, platelet, tomato pomace extracts, extraction process, microencapsulate extract

## Abstract

Aqueous and ethanolic extracts of tomato pomace were examined with the aim of optimizing the extraction process of compounds with cardioprotective activity. Once the results of the ORAC response variables, total polyphenols, °Brix, and antiplatelet activity of the extracts were obtained, a multivariate statistical analysis was performed using the Statgraphics Centurion XIX software. This analysis showed that the most relevant positive effects in the inhibition of platelet aggregation were 83 ± 2% when using the agonist TRAP-6, when the working conditions were the type of tomato pomace conditioning (drum-drying process at 115 °C), phase ratio (1/8), type of solvent (ethanol 20%), and type of extraction (ultrasound-assisted solid–liquid extraction). The extracts with the best results were microencapsulated and characterized by HPLC. The presence of chlorogenic acid (0.729 mg/mg of dry sample) was found, a compound that has a potential cardioprotective effect documented in various studies, in addition to rutin (2.747 mg/mg of dry sample) and quercetin (0.255 mg/mg of dry sample). These results show that the extraction efficiency of compounds with cardioprotective activity depends largely on the polarity of the solvent, thus playing an important role in the antioxidant capacity of the extracts of tomato pomace.

## 1. Introduction

Cardiovascular diseases (CVDs) currently account for nearly half of noncommunicable diseases (NCDs) [1]. Chile, like other countries, has high mortality rates from cardiovascular diseases [2]. CVDs comprise a group of disorders of the heart and blood vessels. Congenital heart disease, coronary heart disease, peripheral artery disease, rheumatic heart disease, deep vein thrombosis, and pulmonary embolism are included in this group of CVDs [3,4].

The complex pathophysiological process involved in CVD includes the participation of platelets [5]. The interaction of activated platelets with dysfunctional endothelium is an important factor contributing to atherothrombosis and the development of CVD [6].

Diet is a determining factor in CVD, so there is a demand for food sources that contribute to the prevention of CVD and a better quality of life [7]. Adopting healthy eating habits will prevent the onset of most diseases, including CVD.

Previous studies related to tomato or tomasa pomace extracts have demonstrated the cardiovascular protective role of a healthy diet [8], which preferably includes fruits and vegetables, with tomato (*Lycopersicum esculentum*) highlighted among the latter.

According to the World Processing Tomato Council, more than 38.7 million tons of tomato destined for processed products were produced at the end of 2021 [9]. In Chile, the processed and exported volumes of industrial tomato for paste, juice, purée, ketchup, and other products in the 2020 season amounted to almost 152 thousand tons [10], generating at least 6 thousand tons of the industrial by-product of the tomato process, called tomato pomace (TP). TP is mainly composed of tomato skin (27%), pulp remains (40%), and seeds (33%) [11]. Dehydrated TP is rich in dietary fiber (25.7%), sugar (25.7%), protein (19.3%), pectin (7.6%), total fats (5.9%), and minerals (3.9%), while the seed mainly consists of oil, protein [12,13], and a high content of antioxidant compounds such as lycopene (73.4 mg/100 g in tomato peel and 13.0 mg/100 g in tomato seed) [12], lutein, quercetin, kaempferol, and β-carotene. These have been shown to have beneficial effects on human health [14,15,16], including a reduction in platelet aggregation in the blood [8].

The dehydration of tomato pomace is a fundamental stage to avoid fermentation and loss of fresh raw materials, increase shelf life, and reduce transportation and storage costs. In recent years, convection drying [14], freeze drying [17,18], spray drying [19], vacuum drying [20], microwave vacuum drying [21,22], infrared radiation drying [23], and osmotic dehydration [21] have been investigated [24].

Conventional techniques for extracting compounds from plant material, such as maceration, infusion, and solid–liquid, among others, require a large processing time and often involve a large number of toxic solvents [14,25]. This has forced the chemical and food industry to look for new extraction methods. These methods, for the most part, use less solvent and energy, and include cavitation-based extraction, supercritical fluid extraction, ultrasonic-assisted extraction (UAE), ultrafiltration, flash distillation, controlled pressure processes, subcritical water extraction, and microwave-assisted extraction (MAE) [14,25,26,27].

MAE is based on the irradiation of electromagnetic waves at two frequencies (915 and 2450 MHz), which cause the movement of molecules by ion migration and dipole rotation, in turn contributing to an increase in temperature which facilitates the diffusion of compounds from the plant material to the solvent. This results in a greater extraction temperature, which generates a quicker mass transfer rate [27]. On the other hand, the principle of UAE lies in the propagation of ultrasound waves through the medium, which causes cell rupture by cavitation, allowing better penetration of solvents into cellular materials and reducing the length of the process. The use of ultrasound offers substantial advantages over conventional extraction methods, due to its ability to improve the penetration of solvents into the cellular material; this improves its release and diffusion, in addition to reducing the duration of the process [26,28].

These qualities present in the components of tomato pomace and their potential benefit for human health suggest the need to analyze the economic feasibility of extracting bioactive compounds of interest and their use in new products with added value as a real alternative application of these by-products.

Considering the above, this research aimed to study the optimization of the process for obtaining cardioprotective products based on tomato pomace. In this optimization, different parameters were considered, such as type of solvent, drying process, drying temperature, type of extraction, and drying temperature. Additionally, we evaluated the antioxidant activity by oxygen radical absorbance capacity (ORAC) and antiplatelet activity by platelet aggregometry.

## 2. Materials and Methods

### 2.1. Chemicals 

Ethanol (>99.8%) was purchased from Merck and type II water was used as an extraction solvent for the solid–liquid extraction process. Thrombin 6 receptor activating peptide (TRAP-6), Adenosine 5′-diphosphate (ADP), and collagen were obtained from Sigma-Aldrich (St. Louis, MO, USA). Sodium citrate vacuum tubes were purchased from Genetics and Technology spa (GENYTEC), Santiago of Chile, Chile. Anti-CD62P-PE, anti-CD61-FITC, and anti-CD63-PE antibodies were purchased from BD Pharmingen (BD Biosciences, San Diego, CA, USA). The HPLC grade solvents used were purchased from Burdick and Jackson (Muskegon, MI, USA). 

### 2.2. Vegetal Material 

Tomato pomace is an agro-industrial waste that is industrially processed to produce tomato concentrate, which is used to formulate products such as ketchup, sauce, puree, and juice. This processing generates a by-product called tomato pomace, which corresponds mainly to peels and seeds that represent 3 to 5% of fresh tomatoes [29]. In previous investigations we worked with this residue (composed of skin, seeds, and pulp). The tomato pomace, regardless of the variety used, maintains its stable properties after industrial processing [8,30,31,32].

Tomato pomace (TP) was obtained from the Agrozzi tomato pulp extraction plant in the Maule region, Chile. The samples were collected between February and March 2021. 

### 2.3. Tomato Pomace Stabilization 

The stabilization process was carried out through two different processes: vacuum microwave drying and drum drying, as described below:

(a) Drum-drying process: This process was carried out in a double tube drum dryer and applied to viscous pasty or pureed foods, such as pre-gelatinized starches and mashed potatoes [33]. In this process, the feeding of the tomato pomace falls in the space between tubes (0.7 mm of space), giving a contact surface of 1 m^2^ in which thermal exchange takes place. The tomato pomace load was equivalent to 30% of the surface. The stainless-steel drums were heated with saturated steam at 2.5–3.0 bar, causing the drums to heat up to between 115 and 135 °C. The rotation speed of the drums was 0.5 rpm, which implies a residence time of the tomato pomace of 90 s. Tomase was dried in the drum dryer at two different temperatures of 115 and 135 °C.
(b) Vacuum microwave drying process: The use of microwaves in food processing has increased considerably in the last 2 decades and is classified as an advanced thermal technology for food processing. The massive warming phenomenon is the unique feature of these techniques, which reduces the food processing time and improves the quality of the product [34]. Fresh tomase was fed to the vacuum microwave dryer in two batches of 36 and 38.5 kg. The operating variables used were the following: temperature of 60 °C, working pressure in vacuum of 780 MPa, and time of 2.5 h.

### 2.4. Solid–Liquid Extraction Process

To determine which type of stabilization and extraction has the greater effect on the inhibition of platelet aggregation, ORAC, and total polyphenol content, a factorial design was used considering two extraction methods, three TP/solvent ratios, five different solvent mixtures, and considering different polarities: (a) water; (b) water/ethanol 5% (5:95, *v*:*v*); (c) water/ethanol 10% (10:90, *v*:*v*); (d) water/ethanol 15% (15:85, *v*:*v*); and (e) water/ethanol 20% (20:80, *v*:*v*). All these tests were carried out in a time of 60 min. Table 1 shows a summary of the variables that were studied.

The response variables to be evaluated were the following: total soluble solids (°Brix), antioxidant capacity, ORAC (µmol Trolox equivalent g^−1^), total phenols (mg EAG g^−1^), and platelet aggregation inhibition (%). 

#### 2.4.1. Microwave-Assisted Extraction

Dehydrated TP was weighed on a balance (ABT 320-4NM, Kern, Balingen, Germany). It was then placed in a 250 mL beaker. Different ratios R of sample/extraction solvent were used: 1/5, 1/8, 1/10. The samples were placed in contact with distilled water and water–ethanol mixtures at different concentrations: 5%, 10%, 15%, and 20% ethanol *v*/*v*. A domestic microwave (WMW606ADWC Whirlpool, China) having 1200 W power was used. The extraction time was 60 min at power 1, equivalent to 10% (120 W).

#### 2.4.2. Ultrasound-Assisted Extraction

On a balance (Kern ABT 320-4NM, Balingen, Germany), the dehydrated tomase was weighed on a salt scale, then it was placed in a 250 mL beaker. Different phase ratios were used: 1/10, 1/8, and 1/5. The samples were placed in contact with distilled water or with water–ethanol mixtures at different concentrations: 5%, 10%, 15%, and 20% *v*/*v*. An ultrasound bar (QSonica Q125, Newtown, Connecticut, USA) was used, with 50% amplitude (10 kHz) for 60 min at room temperature (20 °C), for which the beaker was placed in a thermoregulated bath.

#### 2.4.3. Preparation of Extracts for Analysis

TP extracts obtained in the UAE and MAE extraction processes were centrifuged in 50 mL conical tubes in a centrifuge (K2015R Centurion Scientific, United Kingdom) at 3500 rpm for 15 min. Subsequently, the supernatant was separated with Corning Falcon cell filters of 100, 70, and 40 μm to obtain the supernatant. The samples were placed in a freezer at −20 °C for later analysis of ORAC and total phenols. In addition, an aliquot was separated, which was lyophilized to perform platelet aggregation inhibition studies.

#### 2.4.4. Determination of Total Soluble Solids

The °Brix measurement principle is based on the refraction of light created by nature and the concentration of solutes [35]. For the determination of soluble solids, a digital refractometer (Hanna Instruments HI 96801, Italy) was used. On the calibrated refractometer and at room temperature (20 °C), 0.5 mL of filtered supernatant was deposited, and the results was read and expressed as °Brix. The reading procedure was performed in triplicate.

#### 2.4.5. Determination of Total Phenols

The total phenol content was determined by the Folin–Ciocalteu method. This method quantifies the reducing power of phenolic compounds on the Folin–Ciocalteu reagent, through the formation of a blue complex that is read at 750 nm [36]. The methodology used is based on OIV-MA-AS2-10, Compendium of International Methods of Analysis—OIV Folin–Ciocalteu Index, 2019 edition. Briefly, a calibration curve was derived with different concentrations from a primary solution of 5 g/L of gallic acid. For the reading of the samples, the following procedure was used: in a transparent plate with 96 wells, the reagents were added in each well in the following order: 200 µL of Folin’s reagent, 15 µL of sample, 40 µL of sodium carbonate solution (7.5% *w*/*v*), and 45 µL of MilliQ water; then, the transparent plate was placed in a microplate reader (Synergy HTX Multi-Mode Reader, Biotek, Santa Clara, CA, USA). Absorbance was measured at 750 nm [37,38].

#### 2.4.6. Determination of Antioxidant Capacity

For the analysis of antioxidant capacity by ORAC, a microplate reader (Synergy HTX Multi-Mode Reader, Biotek, Santa Clara, CA, USA) was used. This instrument is capable of evaluating UV-Vis absorbance, fluorescence, and luminescence. For the measurement, black plates with 96 wells were used. Briefly, sodium fluorescein (0.015 mg mL^−1^), AAPH radical solution (120 mg mL^−1^), and Trolox standard solution (100 µM) were prepared with phosphate buffer (75 mM, pH 7). The operating conditions for the final reaction consisted of 50 μL of diluted extract, Trolox standard, or phosphate buffer (blank), 50 μL of fluorescein, and 25 μL of AAPH incubated at 37 °C in the microplate reader. The fluorescence was recorded every 5 min over 60 min, differences of areas under the fluorescence decay curve (AUC) between the blank and the sample over time were compared, and the results were expressed as μM Trolox Equivalents [37,39].

### 2.5. Microencapsulation of TP Extracts

The concentrated extracts by means of a nanofiltration process with a polymeric tubular membrane of 200 Da were microencapsulated through the spray-dry process using a model ET80 device in co-current mode. The process was carried out using a feed rate of 7.2 Hz equivalent to 0.55 L h^−1^, and the extract was atomized by a 2-fluid conical nozzle, thus entering the chamber in the form of a dispersion of fine droplets. A current of hot air was introduced over these droplets, causing the sudden evaporation of surface moisture. Different microencapsulating agents were tested in 4 different combinations: (1) maltodextrin and (2) gum Arabic, evaluating them independently and in combination.

#### Characterization of Microencapsulate Extract

Microencapsulated extraction of phenolic compounds was conducted in a HPLC system (Agilent 1200 series Santa Clara, CA, USA), with a diode array detector (DAD). Separation was achieved using an Intersil ODS-4 C18 column (250 mm long, 4.6 mm i.d., 5 µm particle diameter; GL Science, Tokyo, Japan). The column was kept at 40 °C. The methodology used was a flow rate of 0.7 mL min^−1^ at 25 °C, and the mobile phase gradient was the following: (A) 0.1 formic acid in MilliQ water, pH 2.6, and (B) 100% acetonitrile. Elution gradients used were 87% of A and 13% of B for 16 min; 45% of A and 55% of B for 7 min; 87 of A and 13% of B for 5 min; and finally returned to the initial conditions. Areas were compared to standard curves corresponding to each found phenolic compound.

### 2.6. Studies on Platelet Aggregation

Preparation of human platelets: All procedures were carried out following the protocol approved by the Scientific Ethics Committee (CEC) of the University of Talca, in accordance with the Declaration of Helsinki («Human Experimentation», 1964). Samples were taken from healthy volunteers who, during the previous two weeks, had not used drugs, among others: non-steroidal anti-inflammatory drugs (NSAIDs) and antiplatelets [40]. Samples were collected from the antecubital vein by phlebotomy in 3.2% sodium citrate tubes (Becton Dickinson Vacutainer Systems, Franklin Lakes, NJ, USA). The tubes were centrifuged at 1200 rpm for 10 min to obtain the PRP. The PRP was adjusted to 200 × 10^9^ platelets/L with platelet poor plasma (PPP). The latter was obtained by centrifuging the rest of the sample at 3000 rpm for 10 min. The platelet count was performed in a Hematology Counter (Bayer Advia 60 Hematology System, Tarrytown, NY, USA).

Antiplatelet activity: The antiplatelet activity of the tomase extracts was evaluated in a lumi-aggregometer (Chrono-Log, Haverton, PA, USA) using the turbidimetric method described by Born and Cross, 1963 [41]. Briefly, 480 μL of PRP (200 × 10^9^ platelets/L) was incubated with the tomase extracts (1 mg/mL) (Alarcón et al., 2015). The vehicle used, negative control, was the solvent in which the extracts were made. Platelet aggregation was stimulated with the addition of agonists (ADP 4 µM and TRAP-6 5 µM). The extracts with the highest antiplatelet activity were selected to study the effect of concentration on antiplatelet activity. Measurements were taken in 6 volunteers and the results of platelet aggregation (percentage of aggregation) were determined by the AGGRO/LINK 8 software. Platelet inhibition was calculated as: [42].

(1) $$Platelet\;aggregation\;inhibition\left(\%\right)=100-\frac{Platelet\;aggregation\;of\;Tomato\;pomace\;extracts}{Platelet\;aggregation\;of\;negative\;control}*100$$

Antiplatelet studies were continued with the most active extracts.

### 2.7. Statistical Analysis

The statistical software StatGraphics Centurion XIX (Statgraphics Technologies, The Plains, VA, USA) was used to perform a multifactorial analysis of variance (ANOVA), to establish the differences between the variables of extraction technology, stabilization type, relationship of phases, and solvent.

The data obtained are presented as the mean ± standard error of the mean (SEM) of the individual experiments and analyzed using Prism 6.0 software (GraphPad Inc., San Diego, CA, USA). Platelet inhibition results were analyzed by ANOVA and Tukey’s post-hoc test to determine the significant differences between samples [43].

## 3. Results and Discussion

### 3.1. Vegetal Material Characterization

The humidity of the collected samples of tomato pomace was measured and found to be 72.65%, as measured using a gravimetric method. In addition, a proximal chemical analysis was carried out on the fresh TP, obtaining the following results: total protein 16.8%; ashes 4.7%; crude fiber 42.9%; fat with acid hydrolysis 12.7%; calcium 0.120%; copper 8.990 mg/kg; total phosphorus 0.440%; iron 54.390 mg/kg; sodium 0.0220%; zinc 22.54 mg/kg. Later, the samples were frozen at −20 °C until stabilization. The results correspond to what was previously reported by Del Valle et al. [44].

### 3.2. Tomato Pomace Drying

Two types of TP drying were evaluated: vacuum microwave drying and rotary drum drying (with two different temperatures of 115 and 135 °C). This drying process was carried out due to the high moisture content of the fresh TP (72.65%), thus avoiding its deterioration.

#### Drum-Drying Process

Table 2 shows the operating conditions and the results of the physicochemical parameters of each of the batches studied using the drum-drying process. It should be noted that the operational performance is defined as the amount of product obtained per amount of raw material entered, while the drying performance is defined as the percentage of the ratio of the of dry solids obtained (product) per quantity of total solids loaded.

From the humidity data as a function of time obtained in the drum-drying process, it is possible to deduce that the drying kinetics were strongly influenced by temperature, since the use of a temperature of 135 °C reduced the time to plateau from 25 to 15 min. That is, for the same process time, the higher temperature used, the higher the drying speed. In addition, it was observed that the loss of water was faster at the beginning of the process that used a temperature of 135 °C, due to the assumption that water steam migrates through pores in the film from the evaporation front inside the film towards the surface of the film [45]. This indicates that temperature is a variable that greatly influences the drying process, an effect than can be attributed to the increase in the convention coefficient of heat transfer.

Vacuum microwave drying: This technique allows working using low temperatures due to heating by microwave penetration, combined with the environment of the vacuum chamber, minimizing the loss of nutrients, and avoiding contact of oxygen with the raw materials, thereby reducing the effect of oxidation. Thus, it is a technology that not only offers better working times, but also allows good quality production. Table 3 shows the operating conditions of this drying process.

The mass of fresh TP (69.8% humidity) conditioned by this process was 77.5 kg, and 14.7 kg of dry tomato pomace was obtained with a final humidity of 5.7%. It should be noted that the initial moisture of the product was determined by the gravimetric method and the final moisture was analyzed using a PCE-MA 110 moisture analyzer.

### 3.3. Total Phenolic Content and Antioxidant Activity of Extracts Tomato Pomace

In this study, the effect of operational variables on the extraction efficiencies of compounds with antiplatelet activity from solid–liquid extractions assisted by microwaves and ultrasound were evaluated. Furthermore, microwave- and ultrasound-assisted solid–liquid extractions were performed using an experimental design that considered the operating variables shown in Table 1. The results obtained in these experiments on a laboratory scale are shown in Table 4. The UAE27 and MAE25 extracts had the highest content of phenolic compounds (1864.7 ± 79.6 and 1786 ± 151.7 GAE/100 g sample dw, respectively), while UAE13 had the lowest content of phenolic compounds (181.2 ± 2.9 GAE/100 g sample dw).

#### 3.3.1. Effect of Extraction Solvent

The antioxidant capacity evidenced in the ORAC assay depends mostly on the solvent used, since the efficiency of the extraction process depends largely on the polarity of the solvent; polarity also affects the interaction of antioxidants with other compounds, which will also play an important role in the resulting antioxidant capacity [46]. Water has a higher polarity than ethanol, which should indicate that it is a better solvent. The MAE16 and UAE 42 extracts had higher antioxidant potential according to the ORAC assay performed (111,743.2 ± 701 and 102,166 ± 2691 µmol ET/100 g dw, respectively); both extracts were obtained by extraction with water/ethanol. The MAE25 extract had lower antioxidant activity, 2440.2 ± 142 µmol ET/100 g dw; the extraction in this case was carried out with water.

In the particular case of the UAE, the use of water–ethanol mixtures also reduces the amount of oxidative compounds resulting from the decomposition of water in the extraction process [47]. This is due to the fact that not only water enters the bubbles resulting from the action of ultrasound in the medium, but also the mixture of ethanol and water enters because ethanol has more stable homolytic bonds, which translates into a lower generation of oxidative compounds [48]; for this reason, the level with the highest percentage of ethanol present in the solvent was found to be the most efficient.

According to Routray and Orsat [49], heating using microwave energy is based on the direct effect of microwave waves on molecules by ionic conduction and dipole rotation, with ionic conduction being the electrophoretic migration of ions when an electromagnetic field is applied, while dipole rotation means the realignment of dipoles with the applied field at 2450 MHz, which is the frequency used in commercial systems; the dipole aligns and randomizes 4.9 × 10^9^ times per second, and this force results in heating. The resistance of the solution to this flow of ions results in friction and this heats the solution.

Ethanol, compared to water, has less ability to obstruct microwave waves as they pass through it, due to its lower dielectric constant, but a greater ability to transform energy into heat. In addition, ethanol reaches higher temperatures than water; this improves the efficiency and speed at which the extraction occurs [50]. This could explain why water with 20% ethanol is better than treatments with a lower percentage of ethanol, since this solvent mixture would conduct heat more efficiently towards the solute and would also reach higher temperatures, contributing in part to the extraction efficiency of the compounds of interest. Similar behavior was reported by Zhang et al. [51], where the assisted extraction of antioxidants (quercetin and rutin) from Euonymus alatus stems was studied using EAM and EAU and different concentrations of ethanol, where it was shown that the extraction yield increased when the ethanol concentration increased from 30% to 50%.

#### 3.3.2. Effect of Mass–Volume Ratio

From Table 5, it is possible to identify that, in most cases, the results show the tendency to decrease the effect of the extraction according to the phase relationship of each treatment, where 1:10 is greater than 1:8, and these two are greater than 1:5. As for conventional extraction, a larger volume of solvent can dissolve the bioactive ingredients of interest more effectively and lead to a higher extraction yield [51,52]; the same effect was reported by some authors in EAM [53,54]. Eskilson and Björklund [55] concluded that the proportion of the sample in the extraction solution should not exceed 30–34% mass–volume; otherwise, the extraction is affected and the efficiency declines.

### 3.4. Effect of Operating Variables

To evaluate the effect of the variables on the ORAC, total phenolic compounds, and platelet inhibition shown in Table 4, a multivariate statistical analysis was performed using the StatGraphics software, using a 95% confidence level. In the statistical model generated by the StatGraphics software, the regression coefficients of each effect were obtained by regression of the experimental data, and the standard errors based on the total error considering 47 degrees of freedom. Table 5 shows the magnitude of the coefficients of the statistical model for the experimental data.

**Figure 1:**
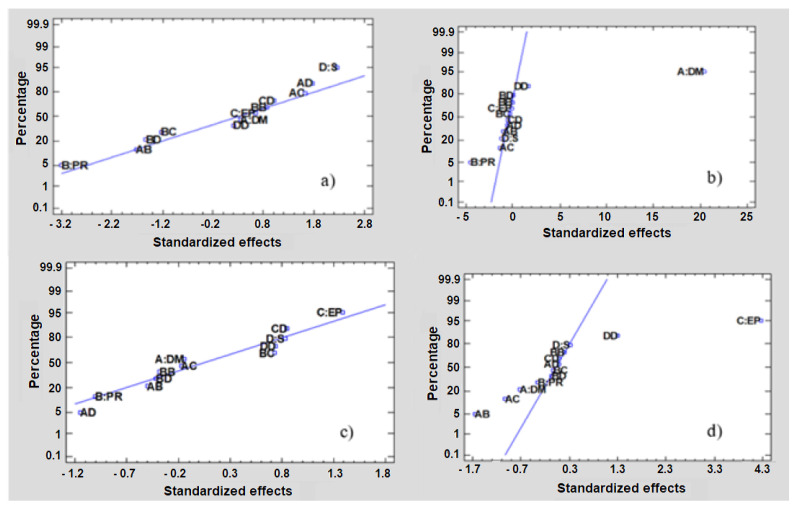
Normal probability plot for (**a**) ORAC; (**b**) total polyphenols, (**c**) platelet inhibition%, TRAP-6 10 µM, and (**d**) platelet inhibition%, ADP 4 µM.

From Table 5, the estimated factors for the ORAC antioxidant activity indicate that the phase relationship is the work variable that matters the most, followed by the type of solvent used; on the other hand, when studying the effect on the content of total polyphenols, it is possible to mention that the most important effect is the drying method, with drum drying at 115 °C being the one that provides the highest values. In the case of platelet inhibition with TRAP-6 and ADP, the effects that most influence maximizing this response are the type of solvent used; this can be seen in the normal probability graph shown in Figure 1.

### 3.5. Studies on Platelet Aggregation of Extracts Tomato Pomace

**Figure 2:**
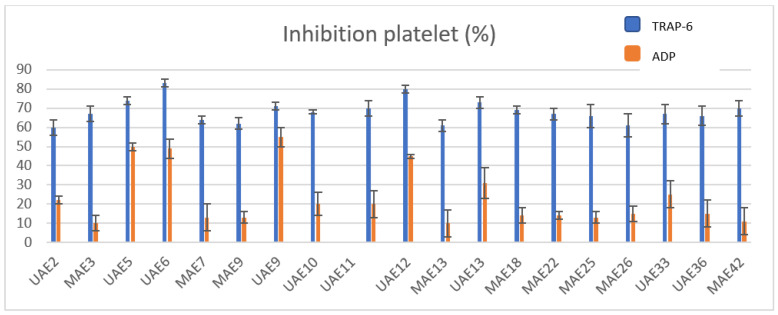
Platelet inhibition of more active extracts of tomato pomace. Platelet aggregation with TRAP-6 and ADP. PRP was preincubated for 5 min with tomato pomace extracts and then stimulated with the indicated agonist. Results are expressed as mean ± SEM, n = 6.

The extracts obtained by UAE showed greater antiplatelet potential than the extracts obtained by MAE (Table 1). This effect was greater when platelet aggregation was stimulated by the platelet thrombin receptor, TRAP-6. Of the 20 extracts with the lowest antiplatelet potential, 13 were obtained by MAE. The 3 extracts that showed the highest activity were UAE 6, UAE 12, and UAE 5 (83 ± 2%, 80 ± 2%, and 74 ± 2%, respectively). On the other hand, the percentage of ADP-stimulated platelet inhibition of these extracts was between approximately 45 and 50%. The ethanolic extracts had the highest activity; additionally, both UAE 6, UAE 12, and UAE 5 were obtained by the drum-drying process at a temperature of 115 °C, and the phase ratio was variable (1/8, 1/10 and 1/5), while the percentage of ethanol varied between 10 and 15%, Figure 2.

### 3.6. Microencapsulation of More Active Extracts

The tomato pomace extract with better response of antiplatelet activity (UAE 6, shown in Table 4) was microencapsulated through the spray-dry process using a model FT80 device in co-current mode. This drying process is widely used in the food industry for the development of various products. Different microencapsulating agents were tested in four different combinations: (1) maltodextrin and (2) gum Arabic, evaluating them independently and in combination. The initial humidity of tomato pomace extracts was 93.3% and the concentration of solids was equivalent to 6 °Brix. To carry out the microencapsulation tests, the encapsulating agent was added until reaching at least 10 ºBrix, which is a minimum concentration of soluble solids for the product to be representative and feasible according to previous experiences. Table 6 shows the formulation of each of the feasible tests. Specifically, trials 1 and 2 independently added maltodextrin and gum Arabic to 5% of the heavy pomasa mass. On the other hand, test 3 mixes both encapsulates in an equivalent way, adding 5% of the pomasa mass and, finally, test 4 seeks to reduce the maltodextrin content, so that gum Arabic was the main added agent.

All the microencapsulation tests evaluated with maltodextrin and gum Arabic were feasible, that is, a dry and stable powdered product was obtained. The operating yield is around 6%, while the humidity of the sprays converges to 3%. By way of observation, the test that presented some inconvenience to go through the feeding line to the drying and subsequent spraying was test 4, which is attributable to its observable difference in rheology compared to the other three tests.

Table 7 shows the results of platelet inhibition of microencapsulated tomase extract with gum Arabic, maltodextrin, and their mixtures. From these results it is possible to observe a decrease in activity, which can be attributed to the dilution in the extract generated by the addition of the encapsulating agent.

On the other hand, the extract and the four samples of encapsulated extracts were analyzed by HPLC to identify the phenolic compounds they contain; the areas obtained were compared with pattern curves corresponding to each phenolic compound previously found. Areas were compared to standard curves corresponding to each found phenolic compound; results are shown in Table 8. With respect to synaptic and coumaric acids, it is possible to see their presence, but their area is under the calibration curve, so it was not possible to quantify them. The UAE6 extract has a higher content of phenolic compounds than the four samples of encapsulated extracts. Trial 4 had the lowest content of chlorogenic acid and rutin, while the encapsulated gum Arabic had the lowest content of quercetin. UAE6 had six times more chlorogenic acid and quercetin, while the presence of rutin was seven times higher. Compounds such as chlorogenic acid, caffeic acid, ferulic acid, and p-coumaric acid have been previously identified by HPLC in tomatoes and there is evidence that these compounds inhibit platelet aggregation [56].

There is increasing evidence that a high intake of fruits and vegetables is beneficial for the prevention of CVD [57]. A diet low in saturated fat and rich in fruits and vegetables can reduce the risks of new cardiac events by 73% [58]. Hence, great interest has arisen in the search for new products that contribute to improving our health and well-being in general.

The antiplatelet activity of fruit and vegetable extracts widely consumed in Chile and the world has been highlighted, among which the tomato stood out [32]. Research has shown that tomato pomace has better antiplatelet potential than even tomato [56]. This underutilized waste makes up about 5% of processed tomatoes and is made up of tomato peel and pulp (78%) and crushed seeds (22%) [8]. Currently this product has some uses (protein supplement for growing lambs, ruminant feed, chemical and nutritional supplements for crackers), which prevents its disposal on agricultural land causing environmental pollution [29].

In this sense, little is known about the effect of the extraction processes on the antiplatelet and antioxidant activity of tomato pomace. We set out to optimize the extraction process of compounds with cardioprotective activity in this by-product. The techniques used involved power, temperature, time, and solvent, thus allowing the improvement in the efficiency of the extraction solvent (water and ethanol) and the reduction in extraction times. The sonication time and extraction solvent have been shown to play a significant role in the antiplatelet activity of some extracts. The different extraction conditions have an effect on the variation in the concentration of the bioactive compounds, as well as on the yield of the extract [29].

Similar to our results, previous studies have linked tomato pomace with both antioxidant and antiplatelet protection, and both activities are associated with a decrease in the prevalence of CVD [30,59]. These effects have been evaluated in vitro and in vivo [60]. Consumption of tomato products was shown to reduce oxidative stress-induced postprandial lipidemia and the associated inflammatory response [30].

The antiplatelet activity of the aqueous extract of tomato pomace observed in vitro is independent of the agonist used (ADP, collagen, TRAP-6, and arachidonic acid) [32]. In vivo experiments show dose-dependent inhibition of platelet aggregation by 8–23%, depending on the agonist used, after 3 h of administration of mainly the tomato extract [61,62,63]. Previous results have also shown that the levels of inhibition of platelet aggregation induced by ADP were different, depending on the ultrasound times, the composition of the tomato pomace, and the solvent used [29], which corresponds to our results. Mechanistic studies suggest that the antiplatelet activity of tomato pomace is mediated by the effect on thrombin receptors (PAR), prevention of p-selectin expression on the surface of platelets, and reduction in GPIIIb/IIa [63].

Research has shown that the cardioprotective effects of tomatoes depend on the presence of bioactive compounds in sufficient quantities to produce a relevant physiological effect [58]. On the other hand, the antioxidant activity of the tomato, as well as the pomace, correlates positively with the content of polyphenols [30], which explains the greater antioxidant activity of the skins compared to the pulp.

Our results, as well as other investigations, show that the extraction process of phenolic compounds, and concentration by lyophilization, allows protection and concentration of bioactive compounds of interest, such as coumaric acid, phloridzin, phloretin, procyanidin B2, luteolin-7-O-glucoside, kaempferol, and quercetin [8]. Other authors have described that tomatoes, and their by-products, contain valuable phytochemicals or bioactive components, mainly flavanones (glycosylated derivatives of naringenin) and flavonols (glycosylated derivatives of quercetin, rutin, and kaempferol) [64].

Our study, in particular, allowed the identification of quercetin, rutin, and chlorogenic acid, known to inhibit platelet aggregation. The cardioprotective potential of quercetin, in particular, as well as its derivatives, has previously been described [65,66]. This flavonol is known to enhance the antiplatelet effects of aspirin [67]. Its mechanism has been related to the inhibition of Fyn and Syk phosphorylation, while promoting the generation of nitric oxide in endothelial cells [66]. This compound inhibits platelet aggregation by blocking GPIIb/IIIa receptors. Quercetin 2 mM significantly prevents the increase in aggregates caused by cardiac infarction [68].

On the other hand, rutin and quercetin rutinoside were identified in our extract. This compound has also stood out because of its antioxidant properties (according to DPPH and ABTS assays) and antiplatelet properties (according to aggregometry) [69]. Rutin also ameliorates stress-induced kidney injury through inhibition of oxidative stress and inflammation by the NOS-mediated NF-κB signaling pathway [70]. It has been noted that the biological potential of flavonoids depends on the number and location of the hydroxyl groups, the presence of the 2,3 double bond in the C ring, the 3- and 5-hydroxy groups, and the glycosylation pattern (C-glycosides or O-glycosides) and position, etc. [69].

Previous work has highlighted the effectiveness of deep eutectic solvents to extract polyphenolic compounds from tomato pomace with the help of ultrasound; specifically, chlorogenic acid was detected as the main phenolic compound in these extracts [71]. While chlorogenic acid has several vascular benefits, such as antiatherosclerosis, antithrombosis, and antihypertensive properties, its antioxidant effects are related to the reduction in LDL oxidation [72]. Additionally, previous studies showed that this acid inhibited the production of ROS by reducing the expression of TRPC1 and, therefore, restored cell viability [72,73]. Dose-dependent chlorogenic acid (0.1 to 1 mmol/L) inhibited platelet secretion and aggregation induced by ADP, collagen, arachidonic acid, and TRAP-6. At these concentrations, it significantly decreased platelet inflammatory mediators (sP-selectin, sCD40L, CCL5, and IL-1β) [72,74], increased intraplatelet cAMP levels/PKA activation, and inhibited thrombus formation in vivo [74]. It also protects endothelial cells from injury by lysophosphatidylcholine (LPC), and consequently inhibits atherosclerosis [72,73].

We can point out that the beneficial effects of a diet rich in fruits and vegetables cannot be attributed to a single compound or a mixture of compounds, but to the synergistic effect of all of them [2]. The compounds identified in the extracts studied can modify platelet activation and/or hemostasis pathways through several mechanisms [58]. In future works, we want to evaluate the mechanism by which the most potent extract exerts its antiplatelet activity. On the other hand, there is a need for more long-term randomized controlled trials, as well as cohort and case-control studies.

## 4. Conclusions

A dietary approach to maintaining cardiovascular health can be considered an important tool in CVD prevention. In this sense, we studied how the different extraction processes influence the cardioprotective potential of tomato pomace. Phenolic compounds possibly responsible for this potential were identified and quantified. The results of our work support the development of promising nutritional strategies involving tomato pomace to address CVD, mediated by antiplatelet and antioxidant effects. Given the large volumes of industrially generated tomato pomace, there is an opportunity to use this by-product to obtain a functional product with antiplatelet and antithrombotic properties that could be useful as an additive in healthy foods, and thus prevent CVD.

## Figures and Tables

**Table 1:** Variables and respective levels used in the experimental test.

Variables	Type of Extraction	Time	TP/Solvent Ratio (R)	Solvent
**Levels**	UAE MAE	60 min 60 min	1/10 1/8 1/5	Water Water/ethanol 5% Water/ethanol 10% Water/ethanol 15% Water/ethanol 20%

**Table 2:** Operating conditions and physicochemical results of batches 1 and 2 dried with drum-drying process.

Operating Conditions	Batch 1	Batch 2
Drum temperature	115 °C	135 °C
Rotation speed	0.5 rpm	0.5 rpm
Initial mass	13.61 kg	12.33 kg
Initial humidity	72.65%	72.65%
Product	3.17 kg	2.96 kg
Product humidity	3.68%	0.41%
Time	0.5 h	0.5 h
Operational performance	23.39%	24.01%
Drying performance	82.03%	87.41%

**Table 3:** Parameters of the tomato pomace drying process using vacuum microwaves.

Parameter	Values
Tomato pomace feed in batch 1 (kg)	38.5
Tomato pomace feed in batch 2 (kg)	36.0
Dry mass obtained (kg)	14.7
Product humidity (%)	5.7
Operating temperature (°C)	60
Vacuum working pressure (MPa)	780
Time (h)	2.5
Operational performance (%)	19.7

**Table 4:** Results of the contents of soluble solids, total phenols, antioxidant activity, and activity antiplatelet of tomato pomace extracts.

Stabilization Process	Sample Name	R	Type of Extraction	Solvent	ORAC µmol ET/100 g dw	Total Phenolic Compounds mg GAE/100 g Sample dw	°Brix	TRAP-6 5 µM		ADP 4 µM	
								PA (%)	Inb (%)	PA (%)	Inb (%)
Drum-dry, 115 °C	MAE 1	1/5	MAE	water	11,788.0 ± 1126	294.3 ± 12.8	4.8 ± 0.05	73 ± 3	15 ± 4	67 ± 3	27 ± 7
Drum-dry, 115 °C	UAE 1	1/5	UAE	water	9021.5 ± 961	268.3 ± 22.5	3.8 ± 0.02	67 ± 3	17 ± 6	47 ± 3	44 ± 4
Drum-dry, 115 °C	MAE 2	1/10	MAE	ethanol 20%	16,654.0 ± 1255	435.5 ± 22.8	7.0 ± 0.05	75 ± 1	8 ± 2	62 ± 1	25 ± 4
Drum-dry, 115 °C	UAE 2	1/10	UAE	ethanol 20%	15,155.5 ± 1396	398.1 ± 34.1	8.8 ± 0.00	32 ± 4	60 ± 4	62 ± 4	22 ± 2
Drum-dry, 115 °C	MAE 3	1/8	MAE	water	10,072.5 ± 49.5	365.7 ± 27.9	2.7 ± 0.08	27 ± 3	67 ± 4	74 ± 3	10 ± 4
Drum-dry, 115 °C	UAE 3	1/8	UAE	water	8941.0 ± 126	371.7 ± 23.6	2.3 ± 0.05	72 ± 5	16 ± 6	71 ± 5	23 ± 1
Drum-dry, 115 °C	MAE 4	1/10	MAE	water	13,227.0 ± 2467	391.8 ± 19.3	3.2 ± 0.04	77 ± 1	9 ± 3	77 ± 1	7 ± 3
Drum-dry, 115 °C	UAE 4	1/10	UAE	water	10,822.0 ± 961	901.6 ± 92.1	2.6 ± 0.08	59 ± 1	30 ± 2	59 ± 1	29 ± 1
Drum-dry, 115 °C	MAE 5	1/5	MAE	ethanol 20%	14,311.0 ± 2358	324.8 ± 4.3	10.8 ± 0.03	80 ± 1	4 ± 2	78 ± 1	7 ± 2
Drum-dry, 115 °C	UAE 5	1/5	UAE	ethanol 20%	9263.5 ± 692	296.5 ± 8.2	10.6 ± 0.00	20 ± 2	74 ± 2	41 ± 2	50 ± 2
Drum-dry, 115 °C	MAE 6	1/8	MAE	ethanol 20%	16,122.0 ± 2397	363.1 ± 19.6	7.70 ± 0.00	59 ± 7	30 ± 9	75 ± 7	18 ± 6
Drum-dry, 115 °C	UAE 6	1/8	UAE	ethanol 20%	13,264.0 ± 622	382.2 ± 36.3	9.40 ± 0.00	13 ± 1	83 ± 2	42 ± 1	49 ± 5
Drum-dry, 115 °C	MAE 7	1/5	MAE	ethanol 5%	9466.0 ± 1199	293.0 ± 6.8	5.6 ± 0.05	29 ± 1	64 ± 2	72 ± 1	13 ± 7
Drum-dry, 115 °C	UAE 7	1/5	UAE	ethanol 5%	8792.5 ± 963.5	317.5 ± 5.3	4.6 ± 0.04	64 ± 3	24 ± 4	72 ± 3	22 ± 2
Drum-dry, 115 °C	MAE 8	1/8	MAE	ethanol 5%	19,544.3 ± 1320	342.2 ± 1.2	3.7 ± 0.00	81 ± 4	10 ± 5	75 ± 4	17 ± 6
Drum-dry, 115 °C	UAE 8	1/8	UAE	ethanol 5%	18,307.6 ± 1123	322.9 ± 3.6	3.9 ± 0.04	58 ± 4	31 ± 5	68 ± 4	26 ± 5
Drum-dry, 115 °C	MAE 9	1/10	MAE	ethanol 5%	22,375.4 ± 987	325.9 ± 7.2	3.1 ± 0.03	31 ± 1	62 ± 3	72 ± 1	13 ± 3
Drum-dry, 115 °C	UAE 9	1/10	UAE	ethanol 5%	19,408.0 ± 1608	400.9 ± 2.9	3.4 ± 0.02	23 ± 1	71 ± 2	37 ± 1	55 ± 5
Drum-dry, 115 °C	MAE 10	1/5	MAE	ethanol 15%	12,035.7 ± 1132	307.3 ± 3.9	8.7 ± 0.00	77 ± 5	10 ± 6	77 ± 5	31 ± 8
Drum-dry, 115 °C	UAE 10	1/5	UAE	ethanol 15%	9770.6 ± 815	294.5 ± 3.9	8.5 ± 0.02	27 ± 1	68 ± 1	66 ± 1	20 ± 6
Drum-dry, 115 °C	MAE 11	1/8	MAE	ethanol 15%	18,374.8 ± 1529	424.4 ± 4.7	6.3 ± 0.03	73 ± 8	19 ± 9	78 ± 8	13 ± 4
Drum-dry, 115 °C	UAE 11	1/8	UAE	ethanol 15%	15,656.6 ± 989	405.3 ± 10.7	7.7 ± 0.00	24 ± 2	70 ± 4	66 ± 2	20 ± 7
Drum-dry, 115 °C	MAE 12	1/10	MAE	ethanol 15%	16,819.2 ± 1263	414.3 ± 6.4	5.7 ± 0.04	76 ± 7	16 ± 8	71 ± 7	21 ± 7
Drum-dry, 115 °C	UAE 12	1/10	UAE	ethanol 15%	14,978.1 ± 145	420.4 ± 5.7	7.4 ± 0.05	15 ± 2	80 ± 2	45 ± 2	45 ± 1
Drum-dry, 135 °C	MAE 13	1/5	MAE	ethanol 5%	6113.1 ± 787	278.3 ± 1.8	6.0 ± 0.05	32 ± 2	61 ± 3	74 ± 2	10 ± 7
Drum-dry, 135 °C	UAE 13	1/5	UAE	ethanol 5%	9722.9 ± 353	181.2 ± 2.9	4.4 ± 0.02	23 ± 3	73 ± 3	63 ± 3	31 ± 8
Drum-dry, 135 °C	MAE 14	1/8	MAE	ethanol 5%	13,875.1 ± 294	441.8 ± 7.1	4.1 ± 0.03	44 ± 1	47 ± 3	79 ± 1	3 ± 1
Drum-dry, 135 °C	UAE 14	1/8	UAE	ethanol 5%	15,107.9 ± 181	412.8 ± 3.6	4.3 ± 0.04	70 ± 4	16 ± 3	72 ± 4	12 ± 5
Drum-dry, 135 °C	MAE 15	1/10	MAE	ethanol 5%	16,723.6 ± 695	712.7 ± 6.4	3.5 ± 0.03	36 ± 5	58 ± 7	70 ± 5	24 ± 4
Drum-dry, 135 °C	UAE 15	1/10	UAE	ethanol 5%	13,611.1 ± 702	434.0 ± 14.9	3.7 ± 0.04	36 ± 5	58 ± 7	70 ± 5	24 ± 4
Drum-dry, 135 °C	MAE 16	1/5	MAE	ethanol 10%	111,743.2 ± 701	291.1 ± 1.1	7.9 ± 0.07	78 ± 5	7 ± 3	84 ± 5	1 ± 0
Drum-dry, 135 °C	UAE 16	1/5	UAE	ethanol 10%	10,012.6 ± 696	306.9 ± 4.3	7.8 ± 0.06	56 ± 6	43 ± 5	44 ± 4	56 ± 7
Drum-dry, 135 °C	MAE 17	1/8	MAE	ethanol 10%	24,836.3 ± 399	384.8 ± 9.5	5.7 ± 0.05	85 ± 2	0 ± 0	85 ± 2	0 ± 0
Drum-dry, 135 °C	UAE 17	1/8	UAE	ethanol 10%	11,742.8 ± 39	411.5 ± 9.6	5.9 ± 0.00	46 ± 5	38 ± 7	60 ± 5	18 ± 4
Drum-dry, 135 °C	MAE 18	1/10	MAE	ethanol 10%	22,505.1 ± 796	405.9 ± 9.9	5.2 ± 0.00	27 ± 2	69 ± 2	79+ ± 2	14 ± 4
Drum-dry, 135 °C	UAE 18	1/10	UAE	ethanol 10%	14,229.2 ± 2242	645.9 ± 9.3	5.6 ± 0.03	85 ± 2	0 ± 0	86 ± 2	0 ± 0
Drum-dry, 135 °C	MAE 19	1/5	MAE	ethanol 15%	15,800.5 ± 362	284.9 ± 5.0	9.1 ± 0.05	64 ± 6	22 ± 9	72 ± 6	13 ± 5
Drum-dry, 135 °C	UAE 19	1/5	UAE	ethanol 15%	9838.3 ± 48	278.9 ± 4.9	8.5 ± 0.05	54 ± 2	36 ± 1	50 ± 2	40 ± 3
Drum-dry, 135 °C	MAE 20	1/8	MAE	ethanol 15%	16,165.4 ± 110.4	383.2 ± 11.2	7.2 ± 0.05	59 ± 4	26 ± 4	67 ± 4	8 ± 6
Drum-dry, 135 °C	UAE 20	1/8	UAE	ethanol 15%	13,161.3 ± 0.6	413.2 ± 4.2	6.9 ± 0.00	66 ± 6	22 ± 8	57 ± 6	38 ± 3
Drum-dry, 135 °C	MAE 21	1/10	MAE	ethanol 15%	16,266.8 ± 1550	444.2 ± 9.2	6.5 ± 0.05	36 ± 3	55 ± 3	76 ± 3	2 ± 5
Drum-dry, 135 °C	UAE 21	1/10	UAE	ethanol 15%	14,421.5 ± 552	469.1 ± 2.1	7.3 ± 0.05	55 ± 1	35 ± 1	60 ± 1	28 ± 3
Drum-dry, 135 °C	MAE 22	1/5	MAE	ethanol 20%	11,567.8 ± 752	600.9 ± 13.3	10.1 ± 0.00	25 ± 2	67 ± 3	70 ± 2	14 ± 2
Drum-dry, 135 °C	UAE 22	1/5	UAE	ethanol 20%	9093.1 ± 516	248.9 ± 9.6	9.6 ± 0.08	35 ± 3	56 ± 3	63 ± 3	16 ± 4
Drum-dry, 135 °C	MAE 23	1/8	MAE	ethanol 20%	15,958.7 ± 534	406.3 ± 5.9	8.3 ± 0.00	78 ± 4	9 ± 5	79 ± 4	15 ± 2
Drum-dry, 135 °C	UAE 23	1/8	UAE	ethanol 20%	11,835.6 ± 617	326.7 ± 20.3	8.6 ± 0.04	69 ± 2	19 ± 0	68 ± 2	18 ± 1
Drum-dry, 135 °C	MAE 24	1/10	MAE	ethanol 20%	19,077.7 ± 33	449.6 ± 20.3	8.6 ± 0.03	56 ± 7	31 ± 7	77 ± 7	15 ± 8
Drum-dry, 135 °C	UAE 24	1/10	UAE	ethanol 20%	18,846.2 ± 1171	444.4 ± 1.4	8.6 ± 0.04	62 ± 8	31 ± 9	69 ± 8	23 ± 2
Drum-dry, 135 °C	MAE 25	1/5	MAE	water	2440.2 ± 142	1786.5 ± 151.7	4.5 ± 0.01	26 ± 4	66 ± 6	71 ± 4	13 ± 3
Drum-dry, 135 °C	UAE 25	1/5	UAE	water	4354.6 ± 92	1410.3 ± 135.9	4.0 ± 0.03	72 ± 4	15 ± 3	71 ± 4	15 ± 1
Drum-dry, 135 °C	MAE 26	1/8	MAE	water	10,774.9 ± 253	1552.8 ± 154.4	2.8 ± 0.00	30 ± 5	61 ± 6	70 ± 5	15 ± 4
Drum-dry, 135 °C	UAE 26	1/8	UAE	water	1,296,979 ± 475	1752.1 ± 63.7	2.6 ± 0.01	66 ± 3	23 ± 4	66 ± 3	29 ± 4
Drum-dry, 135 °C	MAE 27	1/10	MAE	water	18,073.4 ± 67.7	1674.2 ± 36.1	2.0 ± 0.00	73 ± 5	14 ± 6	77 ± 5	16 ± 4
Drum-dry, 135 °C	UAE 27	1/10	UAE	water	17,915.3 ± 1326	1864.7 ± 79.6	2.2 ± 0.00	64 ± 5	29 ± 6	61 ± 5	32 ± 4
Vacuum microwave	MAE 28	1/5	MAE	water	3549.3 ± 544	1270.8 ± 113.6	5.5 ± 0.05	62 ± 3	26 ± 4	65 ± 3	19 ± 4
Vacuum microwave	UAE 28	1/5	UAE	water	6681.1 ± 672.5	1174.4 ± 15.3	4.8 ± 0.00	31 ± 5	59 ± 7	73 ± 5	11 ± 2
Vacuum microwave	MAE 29	1/8	MAE	water	7176.6 ± 547	1383.8 ± 38.5	3.6 ± 0.00	35 ± 2	56 ± 3	72 ± 2	13 ± 4
Vacuum microwave	UAE 29	1/8	UAE	water	9311.8 ± 74	1330.5 ± 114	3.1 ± 0.0	39 ± 2	52 ± 3	69 ± 2	14 ± 4
Vacuum microwave	MAE 30	1/10	MAE	water	13,978.0 ± 798	1408.5 ± 21.4	2.9 ± 0.00	66 ± 7	20 ± 6	73 ± 7	12 ± 5
Vacuum microwave	UAE 30	1/10	UAE	water	15,944.2 ± 130	1385.6 ± 121.6	2.6 ± 0.00	56 ± 3	35 ± 4	50 ± 3	45 ± 6
Vacuum microwave	MAE 31	1/5	MAE	ethanol 5%	6351.4 ± 130	1386.7 ± 42.0	6.9 ± 0.05	51 ± 6	37 ± 5	72 ± 6	6 ± 4
Vacuum microwave	UAE 31	1/5	UAE	ethanol 5%	7044.9 ± 441	1088.6 ± 35.3	6.2 ± 0.05	49 ± 2	50 ± 3	67 ± 2	13 ± 4
Vacuum microwave	MAE 32	1/8	MAE	ethanol 5%	11,489.5 ± 329	1385.4 ± 281.3	4.8 ± 0.00	66 ± 2	20 ± 3	73 ± 6	10 ± 5
Vacuum microwave	UAE 32	1/8	UAE	ethanol 5%	13,511.5 ± 998	1273.7 ± 52.9	4.5 ± 0.01	59 ± 3	32 ± 4	55 ± 3	49 ± 6
Vacuum microwave	MAE 33	1/10	MAE	ethanol 5%	16,343.7 ± 810	1408.5 ± 98.4	3.9 ± 0.00	52 ± 4	34 ± 5	73 ± 4	11 ± 1
Vacuum microwave	UAE 33	1/10	UAE	ethanol 5%	21,065.9 ± 113	1291.8 ± 10.6	3.5 ± 0.00	27 ± 6	67 ± 5	52 ± 6	25 ± 7
Vacuum microwave	MAE 34	1/5	MAE	ethanol 10%	54,529.0 ± 4344	1347.9 ± 40.3	8.2 ± 0.05	83 ± 1	3 ± 2	77 ± 1	16 ± 4
Vacuum microwave	UAE 34	1/5	UAE	ethanol 10%	7269.0 ± 556	1009.6 ± 13.9	7.9 ± 0.02	88 ± 2	0 ± 0	72 ± 2	22 ± 5
Vacuum microwave	MAE 35	1/8	MAE	ethanol 10%	9574.9 ± 703	1291.6 ± 96.0	5.4 ± 0.00	70 ± 5	16 ± 3	72 ± 4	12 ± 5
Vacuum microwave	UAE 35	1/8	UAE	ethanol 10%	12,276.6 ± 106	1128.9 ± 73.2	5.1 ± 0.00	80 ± 4	11 ± 4	75 ± 4	16 ± 6
Vacuum microwave	MAE 36	1/10	MAE	ethanol 10%	18,041.2 ± 454	1442.4 ± 43.9	3.9 ± 0.00	75 ± 1	8 ± 2	62 ± 1	25 ± 4
Vacuum microwave	UAE 36	1/10	UAE	ethanol 10%	18,538.0 ± 863	1232.8 ± 56.8	3.5 ± 0.02	29 ± 4	66 ± 5	70 ± 4	15 ± 7
Vacuum microwave	MAE 37	1/5	MAE	ethanol 15%	12,847.2 ± 590	1440.3 ± 288.7	9.4 ± 0.05	50 ± 5	36 ± 5	75 ± 4	14 ± 2
Vacuum microwave	UAE 37	1/5	UAE	ethanol 15%	12,671.4 ± 937	1075.9 ± 124.1	9.4 ± 0.01	34 ± 3	56 ± 5	73 ± 6	25 ± 4
Vacuum microwave	MAE 38	1/8	MAE	ethanol 15%	17,541.5 ± 1129	1099.2 ± 120.4	7.6 ± 0.02	33 ± 5	57 ± 8	66 ± 5	20 ± 1
Vacuum microwave	UAE 38	1/8	UAE	ethanol 15%	16,816.7 ± 114	1123.4 ± 85.8	7.4 ± 0.05	35 ± 6	57 ± 5	70 ± 6	28 ± 3
Vacuum microwave	MAE 39	1/10	MAE	ethanol 15%	19,179.1 ± 694	1358.5 ± 23.2	4.2 ± 0.02	39 ± 5	53 ± 6	74 ± 5	10 ± 4
Vacuum microwave	UAE 39	1/10	UAE	ethanol 15%	18,036.3 ± 1224	1158.5 ± 43.0	4.0 ± 0.00	73 ± 4	15 ± 5	76 ± 4	18 ± 1
Vacuum microwave	MAE 40	1/5	MAE	ethanol 20%	12,840.8 ± 229	1055.1 ± 89.6	11.3 ± 0.05	68 ± 6	21 ± 7	70 ± 6	10 ± 2
Vacuum microwave	UAE 40	1/5	UAE	ethanol 20%	11,658.7 ± 87	1035.8 ± 88.8	10.8 ± 0.03	71 ± 6	17 ± 7	79 ± 6	14 ± 1
Vacuum microwave	MAE 41	1/8	MAE	ethanol 20%	11,505.4 ± 656	1203.8 ± 93.0	8.8 ± 0.01	70 ± 5	22 ± 6	71 ± 5	21 ± 7
Vacuum microwave	UAE 41	1/8	UAE	ethanol 20%	9906.7 ± 834.7	1167.1 ± 59.9	8.8 ± 0.02	65 ± 7	23 ± 6	71 ± 7	14 ± 7
Vacuum microwave	MAE 42	1/10	MAE	ethanol 20%	16,433.8 ± 265	1481.3 ± 158.9	4.7 ± 0.03	24 ± 2	70 ± 4	73 ± 2	11 ± 7
Vacuum microwave	UAE 42	1/10	UAE	ethanol 20%	102,166.1 ± 2691	1490.9 ± 29.3	4.3 ± 0.00	41 ± 6	54 ± 6	39 ± 6	57 ± 4

Data for content of soluble solids, total phenols, and antioxidant activity are expressed as mean ± SEM, *n* = 3 from at least three independent experiments. Data for activity antiplatelet are expressed as mean ± SEM, n = 6 from at least three independent experiments. Inb (%): platelet aggregation inhibition percentage, PA (%): platelet aggregation percentage.

**Table 5:** Regression coefficients of the factors and standard error estimated by regression of the experimental data.

Effect	Estimated Coefficient	Standard Error	Estimated Coefficient	Standard Error	Estimated Coefficient	Standard Error	Estimated Coefficient	Standard Error
	ORAC		Total Phenolic Compounds		Platelet Inhibition%, TRAP-6 10 µM		Platelet Inhibition%, ADP 4 µM	
Average	13,269.7	2909.6	766.96	43.60	35.19	6.77	17.41	3.37
A	917.3	2765.3	845.82	41.44	−0.93	6.44	−2.33	3.20
B	−10,825.5	3386.3	−229.13	50.75	−7.95	7.88	−1.40	3.92
C	1769.6	2765.4	−3.76	41.44	8.93	6.44	13.67	3.20
D	8841.1	3910.7	−72.84	58.60	7.53	9.10	1.43	4.53
AB	−5838.5	3386.7	−53.94	50.75	−3.95	7.88	−6.50	3.92
AC	4488.6	2765.3	−58.85	41.44	−1.13	6.44	−3.27	3.20
AD	6960.6	3910.7	−35.04	50.60	−10.47	9.10	0.37	4.53
BB	5022.8	5866.0	−2.59	87.90	−5.25	13.65	1.40	6.79
BC	−4171.9	3386.7	−13.63	50.75	5.75	7.88	−0.10	3.92
BD	−7308.0	4789.6	0.55	71.77	−4.65	11.15	−0.35	5.54
CD	3977.2	3910.7	−31.92	58.60	7.33	9.10	0.37	4.5
DD	1312.9	6610.3	160.48	99.05	11.43	15.39	9.95	7.65

Where A: Drum dry; B: Phase ratio; C: Extraction process; D: Extraction process.

**Table 6:** Formulation of samples subjected to spray drying for microencapsulation.

N°	Po (g)	Mx (g)	Ga (g)	SS (Brix)	Pr (g)	Hf (%)	Ro (%)	a_w_
**1**	2114	105.7	0	11.8	136.5	2.0	6.3	0.299
**2**	2108	52.7	52.7	10.8	129.1	2.3	6.0	0.245
**3**	2180	0	109	12.4	129.4	3.6	5.7	0.294
**4**	2594	43.3	86.4	14.0	131	3.1	5.9	0.267

Nomenclature: Po: Mass of tomato pomace extract (g); Mx: Mass of maltodextrin (g); Ga: Mass of gum Arabic (g); SS: Concentration of soluble solids of the pomasa mixture with microencapsulating agents (°Brix); Pr: Mass of product obtained (g); Hf: Product moisture (%); Ro: Operating efficiency, (product mass/fed mass) × 100 (%), and aw: water activity. Condition 1: maltodextrin to 5% of the heavy pomasa mass, Condition 2: gum Arabic to 5% of the heavy pomasa mass, Condition 3: mixes both encapsulants in an equivalent way, adding 5% of the pomasa mass, and Condition 4: seeks to reduce the maltodextrin content, so that gum Arabic was the main added agent.

**Table 7:** Antiplatelet activity of microencapsulated extracts.

Sample	TRAP-6 10 µM		ADP 4 µM	
	PA (%)	Inb (%)	PA (%)	Inb (%)
**UAE6**	13 ± 1	83 ± 2	42 ± 1	49 ± 5
**1**	55 ± 4	31 ± 4	57 ± 4	31 ± 1
**2**	45 ± 1	43 ± 4	46 ± 1	42 ± 2
**3**	32 ± 5	59 ± 7	46 ± 5	30 ± 7
**4**	18 ± 2	76 ± 4	37 ± 2	56 ± 3

Condition 1: maltodextrin to 5% of the heavy pomasa mass, Condition 2: gum Arabic to 5% of the heavy pomasa mass, Condition 3: mixes both encapsulants in an equivalent way, adding 5% of the pomasa mass, and Condition 4: seeks to reduce the maltodextrin content, so that gum Arabic was the main added agent. UAE: ultrasonic extraction.

**Table 8:** Phenolic quantification of TP extracts and their microencapsulations.

Sample	Chlorogenic Acid (mg/g of Dry Sample)	Rutin (mg/g of Dry Sample)	Quercetin (mg/g of Dry Sample)
**UAE 6**	0.729	2.747	0.255
**1**	0.137	0.529	0.05
**2**	0.124	0.432	0.041
**3**	0.143	0.564	0.052
**4**	0.110	0.397	0.047

Condition 1: maltodextrin to 5% of the heavy pomasa mass, Condition 2: gum Arabic to 5% of the heavy pomasa mass, Condition 3: mixes both encapsulants in an equivalent way, adding 5% of the pomasa mass, and Condition 4: seeks to reduce the maltodextrin content, so that gum Arabic was the main added agent. UAE: ultrasonic extraction.

## Data Availability

Not applicable.
